# Global versus Local Conservation Focus of U.S. State Agency Endangered Bird Species Lists

**DOI:** 10.1371/journal.pone.0008608

**Published:** 2010-01-06

**Authors:** Jeffrey V. Wells, Bruce Robertson, Kenneth V. Rosenberg, David W. Mehlman

**Affiliations:** 1 Boreal Songbird Initiative, Seattle, Washington, United States of America; 2 W. K. Kellogg Biological Station, Michigan State University, Hickory Corners, Michigan, United States of America; 3 Cornell Lab of Ornithology, Cornell University, Ithaca, New York, United States of America; 4 The Nature Conservancy, Albuquerque, New Mexico, United States of America; University of Bristol, United Kingdom

## Abstract

The development of species priorities for conservation at local or regional scales (for example, within a state or province) poses an interesting paradox. One the one hand, locally or regionally-derived species priorities may lead to greater interest in and resources directed to biodiversity conservation by local or regional institutions. On the other hand, locally or regionally-derived species priorities could overlook national or global priorities. We assessed U.S. state government agency endangered-threatened bird lists to determine the comparative representation of species of global versus local conservation significance on them. State lists tended to be represented primarily by species of low global risk-low global responsibility (range: 15–100%; mean 51%) and high global risk-high global responsibility (range: 0–73%; mean 35%). In 25 states, more than half of the species on the state lists were in the low global risk-low global responsibility category. Most U.S. state agency lists represent a combined strategy of highlighting species of both local and global conservation significance. Even with this combined local-global strategy, most state lists were predominated by species that represent local but not global conservation significance. Such a strategy could have profound negative consequences for many species that are not formally recognized under national endangered species protections but that are also left off of state-level endangered species lists.

## Introduction

Given the widespread and growing worldwide problem of species declines and extinctions, prioritizing species in need of conservation attention is an essential step in allocating limited financial or human resources. Although many globally threatened species have been identified through standardized ranking systems (e.g. IUCN Red List, NatureServe), the burden of identifying priority species at smaller geographic scales often falls on agencies or institutions operating within political boundaries much smaller than the geographic ranges of most species. The development of species priorities for conservation at local or regional scales poses an interesting paradox. Locally or regionally-derived species priorities may lead to greater interest in and resources directed to biodiversity conservation by local or regional institutions. Ideally, a set of locally derived priorities would, collectively, serve to protect or conserve species that are most vulnerable at the global scale. If, however, locally derived species priorities do not reflect species' range-wide or global priorities, conservation actions may potentially have the undesired effect of promoting local species diversity (within political boundaries) at the expense of global diversity.

**Figure 1:**
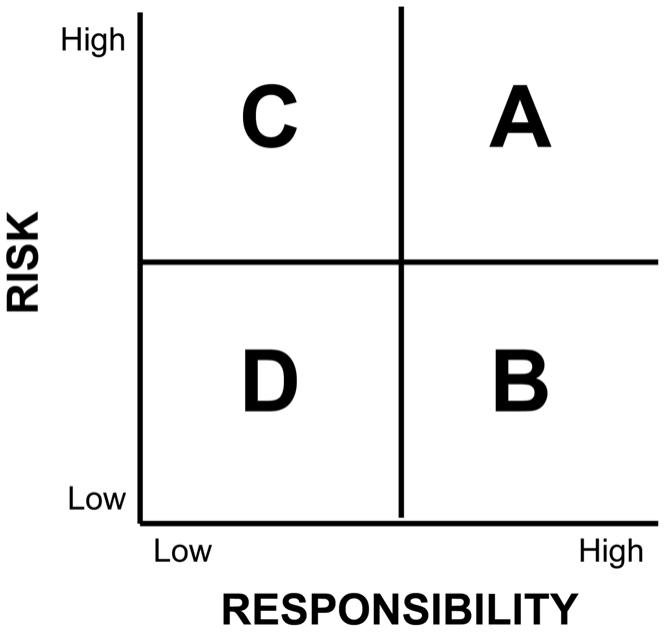
Global conservation risk-responsibility matrix. Each species in a given region can be placed in one of four categories as evaluated against its global extinction risk and the region's responsibility toward sustaining its global population. Species in category A (High Global Risk-High Global Responsibility) are those that are at high global extinction risk and that the region has a high responsibility toward sustaining the global population. Species in category B (Low Global Risk – High Global Responsibility) are those that are at low global extinction risk and that the region has a high responsibility toward sustaining the global population. Species in category C (High Global Risk – Low Global Responsibility) are those that are at high global extinction risk and that the region has a low responsibility toward sustaining the global population. Species in category D (Low Global Risk – Low Global Responsibility) are those that are at low global extinction risk and that the region has a low responsibility toward sustaining the global population.

Two important considerations in developing species conservation priorities are the scale (globally or locally) at which the level of extinction risk is applied [1]–[10] and the biological capacity of a region to contribute towards sustaining populations of a species based on the proportion of the global population that occurs there—a concept that has been labeled “responsibility” [2], [4], [11]–[13]. Each species within a region can be assessed against these two factors. Within a given region, a U.S. state for example, there will be some species that occur there that are of high global extinction risk and some that are of low global extinction risk. There will also be some species for which the state has high global responsibility and some for which the state has low global responsibility. Each species that occurs within a given state can then be placed into one of four categories: high global risk – high global responsibility, high global risk – low global responsibility, low global risk – high global responsibility, and low global risk – low global responsibility (Fig. 1).

In the U.S. there has been widespread development of local and regional conservation capacity, most notably at the state level [14]. In 48 of 50 U.S. states, this has been associated with the development of state endangered, threatened, and/or special concern (E-T-SC) species lists that are used to guide resource, and in some cases, regulatory decisions of government wildlife management institutions [14]–[15]. In a policy review of U.S. state endangered species legislation, George et al. [15] found a wide range of approaches and philosophies embodied in the various state endangered species acts and state government E-T-SC species lists. In interviews with agency staff they found that some state agencies viewed globally or nationally endangered species as the highest priority for state-level conservation while others considered it the state's role to highlight species that were at risk of extinction in their state but not at risk at the global or national level [15].

We explicitly assessed whether these individual state government E-T-SC lists captured more bird species of high or low global conservation risk and bird species for which a state has high or low global conservation responsibility. We chose to use birds for this analysis because they were the only taxonomic group for which a standardized, comprehensive database of conservation scores had been assembled that would allow both global risk and global responsibility to be evaluated for all species in all U.S. states.

Our analyses were limited to describing how U.S. state E-T-SC bird lists represent global versus local scale application of conservation risk and conservation responsibility and did not consider the variety of scientific, political, and socioeconomic factors that may influence the make-up of state ET-SC lists. We do not claim that species of higher global risk or higher global responsibility should necessarily be the highest priority of state lists rather we believe it is important to understand how different states approach the application of concepts of risk and responsibility so that conservation practitioners can consider different options to achieve optimal conservation impact from limited financial resources.

## Methods

We compiled a database of state wildlife agency lists of endangered, threatened, and/or special concern bird species for 47 of the 48 U.S. states that had such lists [15]. We excluded Hawaii because the standardized database we used to generate our risk-responsibility designations (see below) did not include Hawaiian bird species. Because there was great variation among states in whether all three listing categories existed (9 states did not have a special concern category and three states had only a special concern category but not categories for endangered or threatened), we lumped all categories together when evaluating them against the standardized risk-responsibility categories. This approach allowed a more meaningful comparison of conceptual approaches to listing among states as it removed distinctions among listing priorities (endangered versus threatened versus special concern) that are often rooted in policy and politics [15]. Our approach also allowed us to distill each state's priority list to a simple binary variable (listed or not listed) and therefore to make the lists as inclusive as possible, giving more opportunities for more species to appear in each of our four risk-responsibility categories.

To categorize the global risk and global responsibility of each bird species on each state's E-T-SC list we used a database of global conservation assessment scores (available for download at http://www.rmbo.org/pif/pifdb.html) originally developed for use in conservation planning by the Partners In Flight (PIF) conservation coalition [9], [16]–[21]. The PIF species assessment process initially ranks species according to seven criteria reflecting global conservation vulnerability. Five of these criteria are quantitative (breeding and non-breeding distribution, relative abundance, population trend, importance of area), and two (breeding season threats and winter season threats) are based on qualitative assessments by experts [12], [18]–[21]. All assessments go through extensive review at the regional and national level to ensure that they are standardized across species and regions. We note that PIF species assessment scores have been revised since 2000 but changes have been minimal and would have little impact on the overall results of our analysis. Each bird species on each state E-T-SC list was placed into one of our four risk-responsibility categories. Each of the four risk-responsibility categories was derived from scores in the Partners In Flight database as follows.

(A) High Global Risk – High Global Responsibility – Species that are of global conservation concern because of small population size, high rate of decline, or major threats but that have a high proportion of their population in a region so that the region shares significant responsibility for long-term conservation of the species. These are species showing high vulnerability in a number of factors and typically showing significant declines across their range. Species were included in this category for a state if the sum of their seven factor scores for any Bird Conservation Region that overlapped with the state was greater than 21 or if the score was 19–21 and the sum of their Area Importance factor score and Population Trend score was greater than 7 (indicating that the species was declining and was not rare or peripheral in region).

(B) Low Global Risk – High Global Responsibility. – Species that are at low global risk of extinction, but that have a high proportion of their global population in a region so that the region shares significant responsibility for long-term global conservation of the species, even if it is not currently declining or at risk from other factors. These species require long-term planning to ensure healthy and sustainable populations in the region. Species were included in this category for a state if the sum of their seven factor scores for any Bird Conservation Region that overlapped with the state was 19–21, the sum of their Area Importance factor score and Population Trend score was less than 8 and the Percent of Population was greater than 10.

(C) High Global Risk – Low Global Responsibility. – Species of high global conservation concern because of small global population size, high rate of decline, or major threats but that are uncommon or peripheral in a region. Often the remaining populations in that region are threatened, usually because of extreme threats to sensitive habitats. Species were included in this category for a state if the sum of their seven factor scores for any Bird Conservation Region that overlapped with the state was 19–21, the sum of their Area Importance factor score and Population Trend score was less than 8, the Percent of Population was less than 10, and the sum of their Threats Breeding and Threats Non-breeding factor scores was greater than 6 or either Threats Breeding or Threats Non-breeding factors score were 5 (indicating that the species was experiencing extreme threats in the Bird Conservation Region).

(D) Low Global Risk – Low Global Responsibility – Species that are at low risk of extinction at the global level and that have a small proportion of the their global population in a region so that the region has low responsibility for long-term global conservation of the species. Species were included in this category for a state if the sum of their seven factor scores was less than 19 in all Bird Conservation Regions that overlapped with the state.

For each state list we calculated the percent of listed species that occurred in each of the four categories and calculated summary statistics for each of the risk-responsibility categories across all states.

Although the decision-rules used to establish the four categories from these conservation vulnerability scores are arbitrary, they are based on conservation categories established through expert review to capture the same concepts embodied in our risk-responsibility categories as part of the development of PIF bird conservation plans [12]. In preliminary analyses, the higher global risk and higher global responsibility categories (A,B,C, of Fig. 1) were found to capture a higher proportion of the total avifauna of each state (12%–44% of avifauna, average 20%) than occurred on state E-T-SC lists (2%–20% of avifauna, average 8%). Therefore, any bias would be towards state-listed species having more opportunity to be included in one of the higher risk or higher responsibility categories (categories A, B, or C, in Fig. 1). In essence, a species on a state list has more than twice as much chance of being included in one of these higher risk or higher responsibility categories than they would based solely on their representation as part of the total avifauna of that state.

## Results

**Figure 2:**
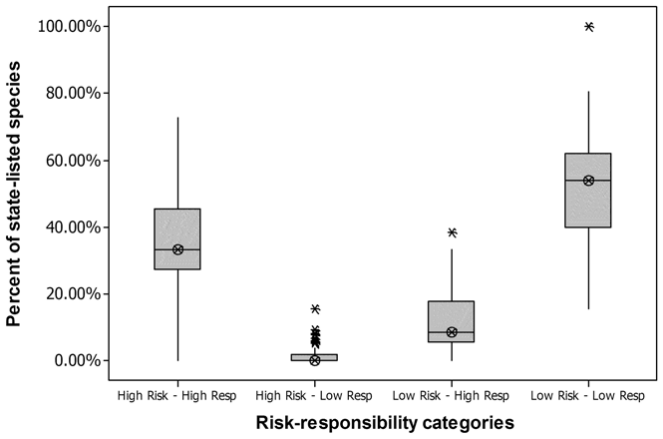
Percent of each state's E-T-SC bird species in each of four risk-responsibility categories. For each category, the boxplot shows mean (horizontal line), 90% quartile (box), 95% quartile (vertical line), and any outliers (asterisks). State E-T-SC lists were predominated by species in the low global risk-low global responsibility category but with significant numbers of species in the high global risk-high global responsibility category.

Most state lists included species in three of the four risk-responsibility categories—high global risk-high global responsibility, low global risk-high global responsibility, and low global risk-low global responsibility. Only 13 (28%) of 47 state lists included any bird species that fell into our high global risk-low global responsibility category. All but one state list included species in our high global risk-high global responsibility category and all state lists included species in our low global risk-low global responsibility category. State lists tended to be represented (Fig. 2) primarily by species in our categories of low global risk-low global responsibility (range: 15–100%;mean 51%) and high global risk-high global responsibility (range: 0–73%; mean 35%). In 25 states (53%), more than half of the species on the state lists were in the low global risk-low global responsibility category. In nine states (19%), more than half of the species on the state list were in the high global risk-high global responsibility category.

## Discussion

The United States is one of relatively few countries where widespread local conservation capacity has developed, particularly within individual states. In most cases, state-level conservation efforts have been associated with the development of locally derived species conservation priorities that are used specifically to guide resource, and in some cases, regulatory decisions of government wildlife management institutions. All 50 U.S. state governments have wildlife management agencies whose combined annual expenditures for non-game wildlife conservation totaled $134,898,266 in 1998 [22]. A number of state governments have also approved large expenditures for land acquisition for conservation purposes. For example, in Florida over $300 million was spent annually for conservation land acquisition from 2000–2003 [23].

Clearly, financial resources of state government institutions directed toward wildlife conservation, though far below estimates of levels needed to preserve biodiversity [22], are being spent based on species priorities developed by state agencies. Species priorities are typically developed locally (within-state) through consultation with wildlife experts who are asked to provide opinions as to which species are at greatest risk of disappearing from the state (e.g., [24]). This process could yield a set of species that collectively reflect national, regional, and local conservation priorities. Alternatively, the process could result in species priorities that highlight mostly species of local concern.

Our analyses show that most U.S. state agency lists of E-T-SC bird species represent a combined strategy of highlighting species of both local and global conservation significance. Even with this combined local-global strategy, most state lists were dominated by species that represent local but not global conservation significance.

Examples of globally secure species that are included on state agency E-T-SC lists include Double-crested Cormorant (*Phalacrocorax auritus* – listed in two states), Great Egret (*Ardea alba* - listed in 12 states), Laughing Gull (*Larus atricilla* –listed in two states), Bank Swallow (*Riparia riparia* - listed in three states), Magnolia Warbler (*Dendroica magnolia –* listed in two states), and Dark-eyed Junco (*Junco hyemalis* - listed in three states). The same pattern was shown in an analysis of state-listed bird species from the northeast and Midwest U.S. with most state lists showing a high representation of species that were locally rare but continentally widespread and abundant [10]. Similarly, Wild et al. [25] found that fern species occurred on U.S. state and Canadian provincial lists in higher proportions than expected and surmised that this was because species status for state/provincial listing is usually evaluated at the local rather than global scale. Bunnell et al. [26] found a conceptually similar situation in British Columbia where less than half of species (of all taxa) of global conservation significance that occur there are listed but 70% of species with peripheral populations are listed.

Locally rare species may represent threatened habitats or potentially demographically and genetically disjunct populations or subspecies [2], [3], [5], [7], [10], [13], [25]–[30] and could represent an early-warning system of regional declines [1], [2], [10], [31]. Some would argue that it is species in this category that are precisely the reason why state-level E-T-SC lists were created and are important.

This mix of strategies across U.S. states and the general tendency to focus more heavily on species of local conservation significance in state E-T-SC lists is not surprising given the variation in endangered-threatened species laws and regulations across states and the often explicit language in legislation that directs state agencies to consider status of species within that state [15], [32]. While few state E-T-SC lists completely exclude species of global conservation significance that occur within their states, there are a number of species that are considered to be of global conservation concern by multiple authorities that are included on only a few state lists. Examples include Lesser Prairie-Chicken (*Tympanuchus pallidicinctus*- listed in one of five states in which it breeds), Long-billed Curlew (*Numenius americanus*-listed in six of 16 states) Bendire's Thrasher (*Toxostoma bendirei*-listed in one of six states), and Golden-winged Warbler (*Vermivora chrysoptera*-listed in 9 of 18 states). Importantly, none of these four high-global-risk species are listed at the federal level under the Endangered Species Act (ESA), and therefore often do not receive any special protections for conservation consideration in the core of their breeding range.

An argument could be made that focusing on globally abundant but locally rare species could promote local diversity at the expense of global diversity since there are always limited resources available for conservation [10], [26], [31], [33]. Since most state E-T-SC lists already include species of local and global conservation significance, there may be flexibility in many state listing processes to broaden the inclusion of species of global conservation significance if desired. For example, New Jersey lists Savannah Sparrow (*Passerculus sandwichensis*), a species of very low range-wide vulnerability but representative of locally threatened grassland habitat within the state, while not listing Saltmarsh Sparrow (*Ammmodramus caudacutus*), a species at risk globally but still common within the state and representative of healthy salt marsh ecoystems. Similarly, Arizona lists Yellow-billed Cuckoo (*Coccyzus americanus*), a species representative of critically threatened riparian habitats within the state but widespread and abundant elsewhere in its range, while not listing Bendire's Thrasher, a globally high-concern species with the bulk of its world population breeding in desert-scrub habitat within the state.

Some have argued that state E-T-SC lists do not necessarily need to include globally threatened species because those species should be listed at the federal level. While this would be the case in an ideal world, the U.S. federal ESA listing does not include many species recognized by numerous scientific listing processes as endangered or threatened. For example, 16 bird species of the continental U.S. and Alaska that appeared on the IUCN Redlist in 2000 were not listed under the U.S. ESA [34]. The conservation implications of U.S. state-level funding and regulatory decisions could be enormous, especially when taken collectively across the multi-state ranges of most species. In the best case, resources and conservation actions of many state governments will be directed toward the recovery of a similar suite of species of national and regional conservation priority. In the worst case, locally rare but continentally abundant and widespread species will receive most conservation resources while species of national and regional conservation priority will be neglected.

This study emphasizes the possibility of a greater role for the prioritization of globally rare, but locally common species on state-level species prioritization lists in the maintenance of biodiversity. We hope these results will be used to strengthen species priority-setting systems for use at local and regional levels across the world. In the U.S., the implications are of particular importance because under the State Wildlife Grants program, all state wildlife agencies were tasked with the creation of statewide Comprehensive Wildlife Conservation Strategies [35] that will guide many of their resource allocation decisions for the immediate future. As part of this process, states developed lists of “species of greatest conservation need,” potentially drawing from both state-endangered and threatened species and priority lists from PIF and other conservation initiatives. The existence of potentially conflicting species lists, derived under different mandates and at different scales, can be confusing to state planners and managers attempting to set priorities for future resource allocation. As state agencies and other local institutions move forward with comprehensive conservation planning that will guide future resource allocations, we urge that they consider, not only species of local conservation significance, but also those of global significance even if those species are relatively abundant within that state (as per recommendations in [36]). More explict consideration of standardized conservation prioritization procedures and global scale species assessment data by state agencies reviewing the species on their lists as well as cooperative multi-state planning could be useful in gaining highest conservation efficacy in each state. In this way state agency listing priorities could represent the full range of conservation needs of species and habitats under their stewardship.
